# Effects of *Clostridium butyricum* on Intestinal Microflora and Metabolism of *Eriocheir sinensis*

**DOI:** 10.3390/ijms241813784

**Published:** 2023-09-07

**Authors:** Xiaoning Gao, Xueting Liu, Yali Wang, Tianwei Wang, Di Fang, Kun Hu

**Affiliations:** 1 National Demonstration Center for Experimental Fisheries Science Education, Shanghai Ocean University, Shanghai 201306, China; 2 National Pathogen Collection Center for Aquatic Animals, Shanghai Ocean University, Shanghai 201306, China

**Keywords:** *Clostridium butyricum*, *Eriocheir sinensis*, intestinal microflora, metabolome

## Abstract

*Clostridium butyricum*, a new probiotic in recent years, can produce butyric acid and short-chain fatty acids. It has the characteristics of strong acid and alkali resistance, high temperature resistance, and strong resistance to most antibiotics, and has more advantages than other probiotics. However, the action mechanism of *C. butyricum* on *Eriocheir sinensis* is still unclear and needs further study. In this study, when *C. butyricum* was added to the basic diet, the number of living bacteria was 0, 1 × 10^6^ and 1 × 10^8^ CFU/g, respectively. The *E. sinensis* were randomly divided into three groups: (blank control group, experimental group 1 (1 × 10^6^ CFU/g) and experimental group 2 (1 × 10^8^ CFU/g)). They were fed an experimental diet for 28 days. The effects of *C. butyricum* on *E. sinensis* were studied by detecting the differences in non-specific immune indexes, intestinal microflora, and metabolites between serum and hepatopancreas. The results showed that *C. butyricum* could improve the antioxidant ability of *E. sinensis* serum and hepatopancreas, protect intestinal tissues, and promote the absorption of nutrients. At the same time, it can enhance the microbial diversity and richness of the *E. sinensis* gut flora. LC-MS metabolomics was used to detect the metabolism of intestinal flora. It was found that *C. butyricum* could up-regulate lysophosphatidylcholine in the intestine. Through the KEGG enrichment pathway, it was found that significantly different metabolites were mainly concentrated in six metabolic pathways. The purine metabolism and alanine, aspartate, and glutamate metabolism pathways showed a downward trend, indicating that the addition of *C. butyricum* to feed could reduce purine metabolism, promote the water-salt balance of the organism’s cells, and reduce inflammation. In this study, it was found that the addition of certain concentrations of *C. butyricum* to feed could improve the antioxidant ability of *E. sinensis*, improve the intestinal flora environment, and promote the growth of beneficial bacteria in the gut. This can promote the body’s metabolism, which is more conducive to its growth.

## 1. Introduction

*Eriocheir sinensis*, commonly known as river crab and hairy crab, is popular with consumers because of its delicious meat and protein-rich qualities [1]. Since the 1980s, when *E. sinensis* was farmed, the *E. sinensis* industry in our country has developed rapidly. With the expansion of the *E. sinensis* farming area and the increasing density, coupled with the deterioration of the surrounding environment, the frequent occurrence of *E. sinensis* diseases has brought huge economic losses to the farmers. *E. sinensis* diseases have seriously hindered the sustainable development of the *E. sinensis* industry. At present, farmers mainly rely on antibiotics for prevention and treatment [2]. Although antibiotic drugs can alleviate the outbreak of bacterial diseases to a certain extent, their abuse can lead to an increase in bacterial resistance [3], which can facilitate the emergence of “Superbugs”. On the other hand, in order to reduce the loss of disease, farmers will increase the dose of antibiotics so that a large number of antibiotics remain in the bodies of farm animals and then in the human body, bringing huge hidden dangers [4]. Therefore, in order to reduce the impact of the abuse of antibiotics, people urgently need to find alternative antibiotics [5]. Probiotics are regarded as the most effective and potential antibiotic substitutes because of their advantages in disease control, improvement of water quality, and intestinal health in farmed animals [6]. At present, it has been reported that probiotics such as *Clostridium butyricum* [7] and *bacillus* [8] have been added to the culture of *E. sinensis*.

*Clostridium butyricum*, also known as *Butyricum butyricum*, is a highly oxygen-sensitive Gram-positive bacteria bacillus that is widely distributed in soil, rivers, animal faces, and the intestines of humans and various animals [6,9,10]. As a kind of beneficial bacteria, *C. butyricum* has attracted much attention in recent years. Studies show that intestinal flora plays an important role in the growth of animals. On the one hand, intestinal flora participates in the digestion and absorption of nutrients; on the other hand, the structure of intestinal flora affects the stability of the animal’s immune system [11]. The imbalance of intestinal flora structure will lead to the decline of immunity and the proliferation of pathogenic bacteria in the intestinal tract, leading to various diseases [12]. *C. butyricum* prevents intestinal disease by promoting the proliferation of beneficial intestinal flora and inhibiting the growth of intestinal pathogens [13]. Duan et al. found that adding *C. butyricum* to the diet could improve the composition of the intestinal microflora of *Penaeus vannamei*, decrease the activity of potential pathogenic bacteria, and increase the activity of beneficial bacteria [14]. Studies by Yuan Hailin et al. found that the addition of *C. butyricum* to diets increased intestinal short-chain fatty acid and nutrient utilization, thereby improving the growth performance of *P. vannamei* [15]. In addition, *C. butyricum* can promote the development of intestinal villi, or microvilli, and improve the structural integrity of the gut, thereby improving the digestion and absorption of nutrients in the gut [16,17]. Zhu Jianguo et al. found in the study of *S. aequifasciata* that adding 1 × 10^5^ CFU/g *C. butyricum* to the diet could increase the height and width of the villi in the midgut, increase the secretion of acidic mucopolysaccharides, improve intestinal digestion, and improve nutrient utilization [18]. But there is a bit of a gap in the mechanism of *E. sinensis*.

In this study, high-throughput sequencing technology was used to detect the intestinal microflora of *E. sinensis*, aiming to explore the regulatory effect of *C. butyricum* on the intestinal flora of *E. sinensis*. And it provides a theoretical basis for the rational utilization of *C. butyricum* in aquaculture. At the same time, the effect of adding *C. butyricum* in feed on the metabolic profile of *E. sinensis* was analyzed by LC-MS metabonomics, and the key differential metabolites were screened by a series of detection methods, then combined with the results of high-throughput sequencing to find the relationship between intestinal flora and metabolism. Further understanding of the relationship between intestinal flora structure and intestinal metabolism will lay the foundation for future research.

## 2. Results

### 2.1. Effect of C. butyricum on Serum Antioxidant Capacity of E. sinensis

The effects of different concentrations of *C. butyricum* on the serum antioxidant capacity of *E. sinensis* are shown in Table 1. From the table, we can see that the serum antioxidant capacity of *E. sinensis* added with *C. butyricum* is higher than that of the control group, and there is no significant difference in the activity of catalase (CAT) or total antioxidant capacity (T-AOC). The activity of glutathione (GSH) in experimental group 2 was significantly higher than that in the control group. For the activity of malonaldehyde (MDA), we can see that the experimental group was significantly lower than the control group (*p* < 0.05).

### 2.2. Effects of C. butyricum on Antioxidant Capacity of Hepatopancreas in E. sinensis

The effects of different concentrations of *C. butyricum* on the antioxidant capacity of the hepatopancreas of *E. sinensis* are shown in Table 2. We can find that the activity of superoxide dismutase (SOD) and T-AOC in the hepatopancreas increased with the increase in the concentration of *C. butyricum*, while the activity of MDA was just the opposite. The activities of CAT and GSH in the 10^8^ cfu/mL *C. butyricum* group were significantly higher than those in the control group (*p* < 0.05).

### 2.3. Effects of C. butyricum on Intestinal Tissue of E. sinensis

The villus length and crypt depth of intestinal tissue structure in the control group and the experimental group are shown in Table 3. With the increase in *C. butyricum* concentration, villus length and crypt depth also increased, and the concentration of 1 × 10^8^ cfu/mL increased significantly (*p* > 0.05).

### 2.4. Effects of C. butyricum on Intestinal Flora of E. sinensis

#### 2.4.1. Sequencing Data Quality Analysis

As shown in Table 4, the raw read data of 12 samples were distributed between 78,223–81,936, and the clean tag data were distributed between 55,334–74,393. After quality control, the number of valid tags (the data for final analysis) was distributed between 54,184 and 73,368. The number of ASVs in each sample was distributed between 119 and 394. The coverage index was 99%, approaching 100%. Therefore, this sequencing can reflect the true situation of the intestinal flora.

#### 2.4.2. Intestinal Microbial Dilution Curve

**Figure 1:**
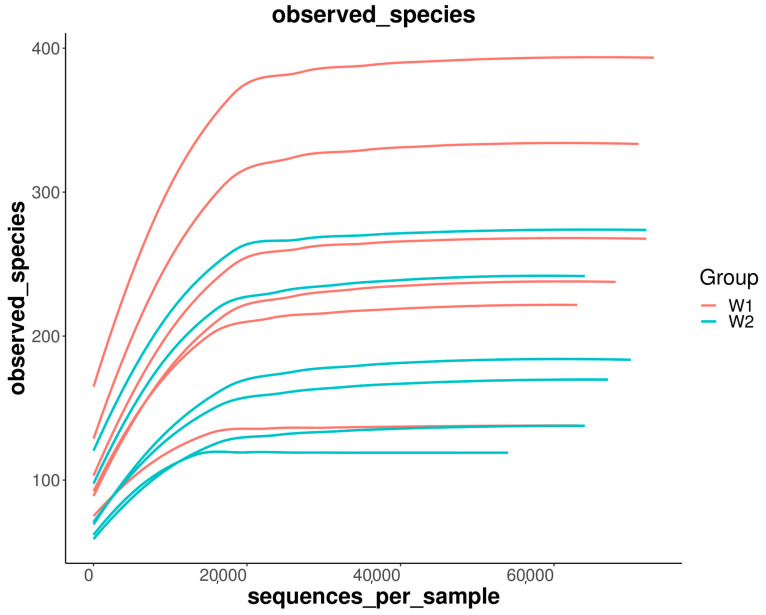
Rarefaction curves of samples.

A rarefaction curve is constructed by counting the number of species represented by a certain number of sequences randomly selected from the samples. As shown in Figure 1, the curve shows a sharp rise followed by a smooth trend to a certain extent as the number of sequencing lines increases. This suggests that a large number of species in the *E. sinensis* gut have been found and will not increase significantly with the increase in the number of sequences, indicating that the sequencing depth has basically covered all species in the sample and the sample sequence is sufficient so that data can be analyzed.

#### 2.4.3. Analysis of Alpha Diversity of Intestinal Flora of *E. sinensis* by *C. butyricum*

**Figure 2:**
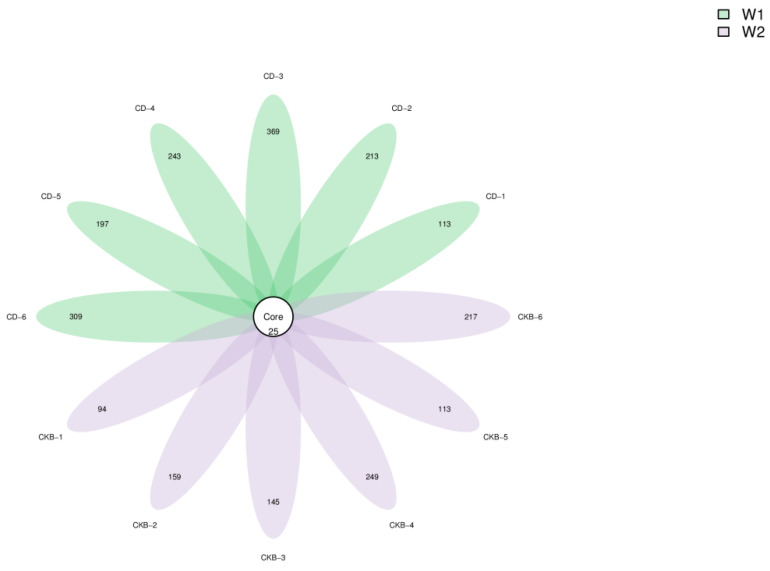
The OUT-distribution petal diagram of the sample.

Operational taxonomic units (OUT) are used in phylogenetic or population genetics studies, where sequences are usually divided into different OTUs according to a similarity threshold of 97%. Each OTU is usually regarded as a microbial species sequence. Petal Figure 2 shows that there were 977 OTUs in the control group and 1444 OTUs in the experimental group. There were 25 OTUs between the parallel groups.

The diversity and richness of individual samples were reflected in alpha diversity. The Ace index and Chao index are usually used to reflect the richness of the species community, and the larger the index value, the higher the species richness. The Shannon index sum is used to reflect the microbial diversity within the community, and the larger the Shannon index value is, the greater the microbial diversity. As shown in Table 5, there was no significant difference between the Ace and Chao indices in the *E. sinensis* microbial diversity indices, but the Shannon index of the experimental group was significantly higher than that of the control group. The results indicated that the addition of 1 × 10^8^ experimental diets could improve the microbial diversity and abundance of *E. sinensis* gut microbiota to a certain extent.

#### 2.4.4. Analysis of Bate Diversity of Intestinal Flora of *E. sinensis* by *C. butyricum*

**Figure 3:**
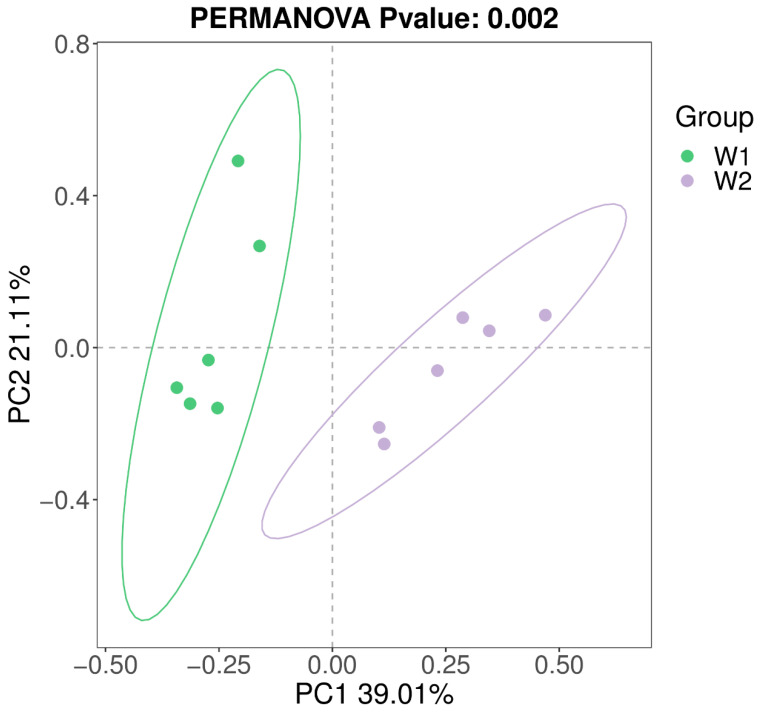
Principal coordinate analysis diagram. Note: Each point in the figure represents a sample, the same color is the same grouping. The closer the samples of the same group are, and there is a significant distance between them and other groups, indicating that the grouping effect is better.

The similarity or difference of the sample community composition was studied by using the non-constrained data dimension reduction method of principal co-ordinate analysis (PCoA). The difference between individuals or groups can be observed by PCoA. As shown in Figure 3, there was no significant aggregation between the experimental group and the control group. That is, there was a large difference in species composition, and the grouping effect was good.

#### 2.4.5. Effects of *C. butyricum* on Intestinal Flora Composition of *E. sinensis*

**Figure 4:**
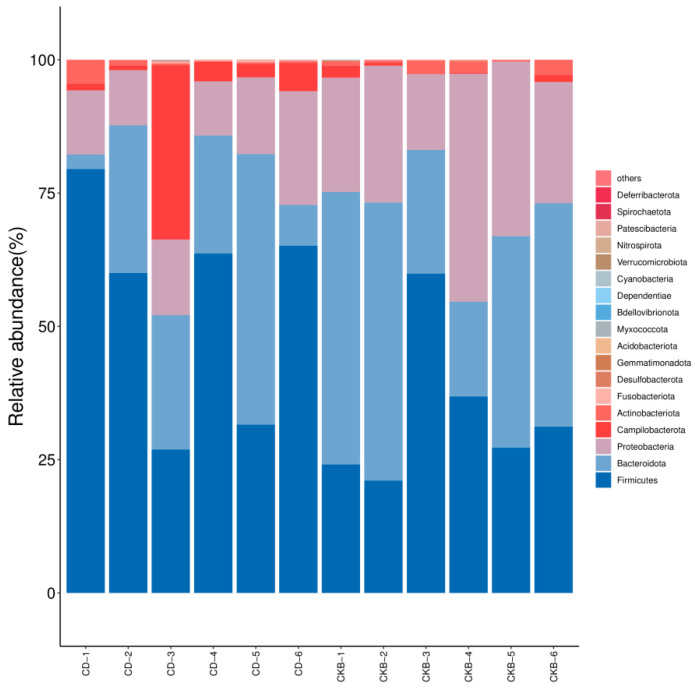
Species distribution (phylum level).

The *E. sinensis* distribution of intestinal microbial species in phyla groups is shown in Figure 4. At the phylum level, there are three dominant microbial communities in the *E. sinensis* gut: *Firmicutes*, *Bacteroidota*, and *Proteobacteria*. The abundance of *Firmicutes* in the intestinal tract of the experimental group was significantly higher than that of the control group, while the abundance of *Bacteroidota* and *Proteobacteria* was significantly lower than that of the control group.

**Figure 5:**
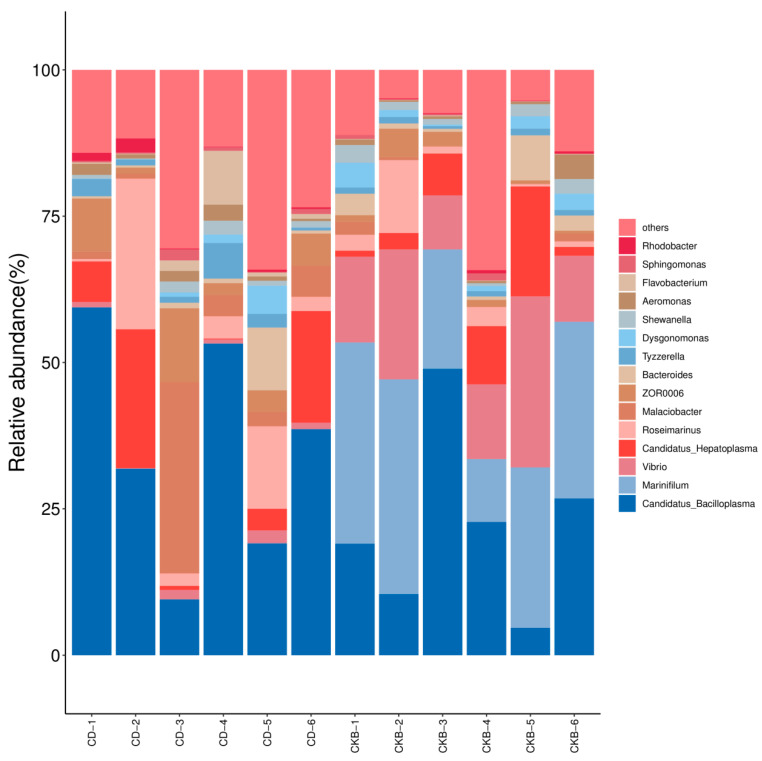
Species distribution (genus level).

At the level of genus classification, it can be seen from Figure 5 that *Candidatus_Bacilloplasma* is dominant in both groups, and the abundance of the experimental group is higher than that of the control group. *Marinifilum* and *Vibrio* were the dominant bacteria in the control group, but their content was very low in the experimental group. *Candidatus_Hepatoplasma* and *Roseimarinus* were the dominant flora in the two groups, and the abundance of *Candidatus_Hepatoplasma* and *Roseimarinus* in the experimental group was significantly higher than that in the control group.

#### 2.4.6. Statistical Analysis of Microbial Multivariate Variables

**Figure 6:**
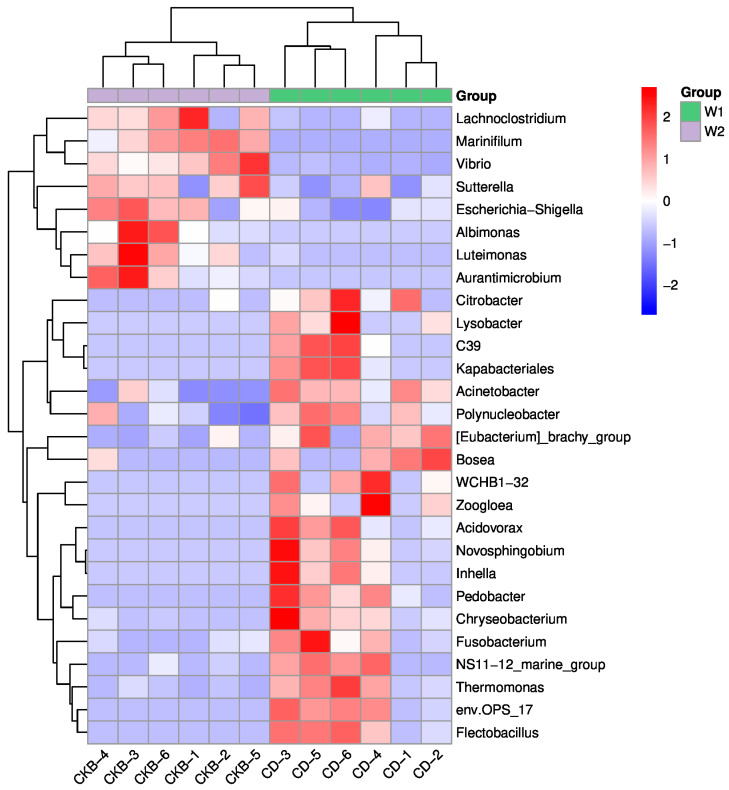
Heatmap of distinct species. Note: The horizontal is the sample information; the vertical is the species annotation information. The cluster tree on the left is the species cluster tree, and the cluster branches above represent samples from different groups. Red indicates higher relative abundance, and blue indicates lower relative abundance.

A *T*-test analysis can be used to find out the significant difference between the experimental group and the control group. A statistical analysis of differences was performed at the genus level, as shown in Figure 6. The main different bacteria genera between the experimental group and the control group were *Marinifilum*, *Vibrio*, *Acinetobacter*, env.OPS_17, etc. The abundances of *Marinifilum* and *Vibrio* in the experimental group were significantly lower than those in the control group, while the abundances of *Acinetobacter* and Env. OPS_17 had higher abundances than the control group.

### 2.5. Effects of C. butyricum on Intestinal Flora Metabolism of E. sinensis

#### 2.5.1. Sample Raw Data and Principal Component Analysis

**Figure 7:**
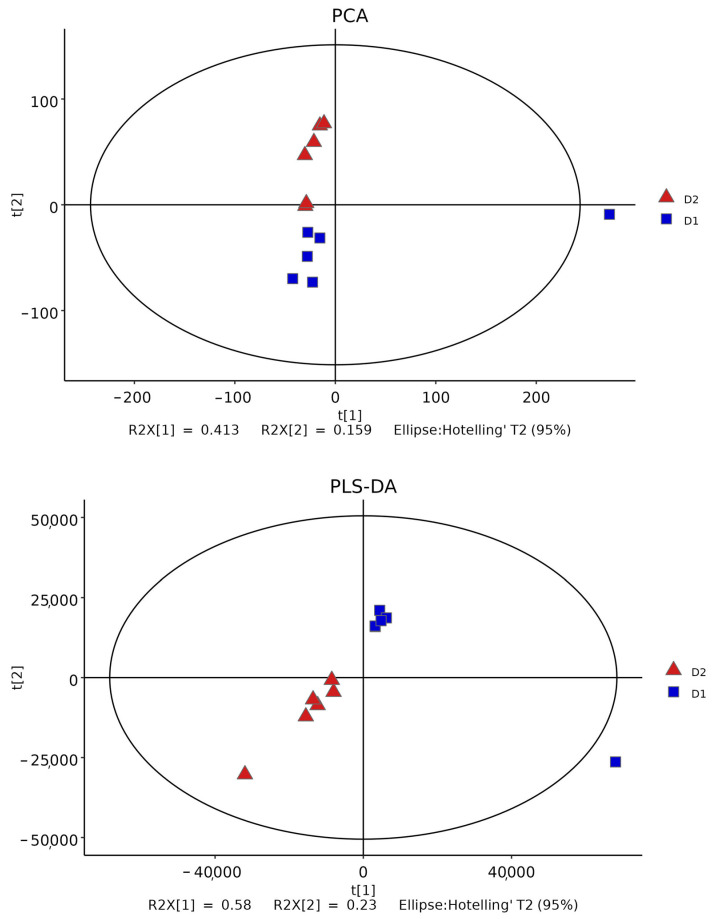
PCA and PLS-DA scores of control group and experimental group.

PCA is a kind of unsupervised analysis that reflects the original situation of the data and is helpful to understand the overall situation of the data and grasp it as a whole. Partial least squares discriminant analysis PLS-DA is a supervised discriminant statistical method that uses partial least squares regression to model the relationship between metabolite expression and sample grouping and predict the sample class. As can be seen from Figure 7, the samples of the blank group and the experimental group are both in the 95% confidence interval and can be separated evenly and completely in space without any overlap. This showed that there were significant differences in metabolites between the two groups, which enabled further analysis.

#### 2.5.2. Screening of Differential Metabolites

**Figure 8:**
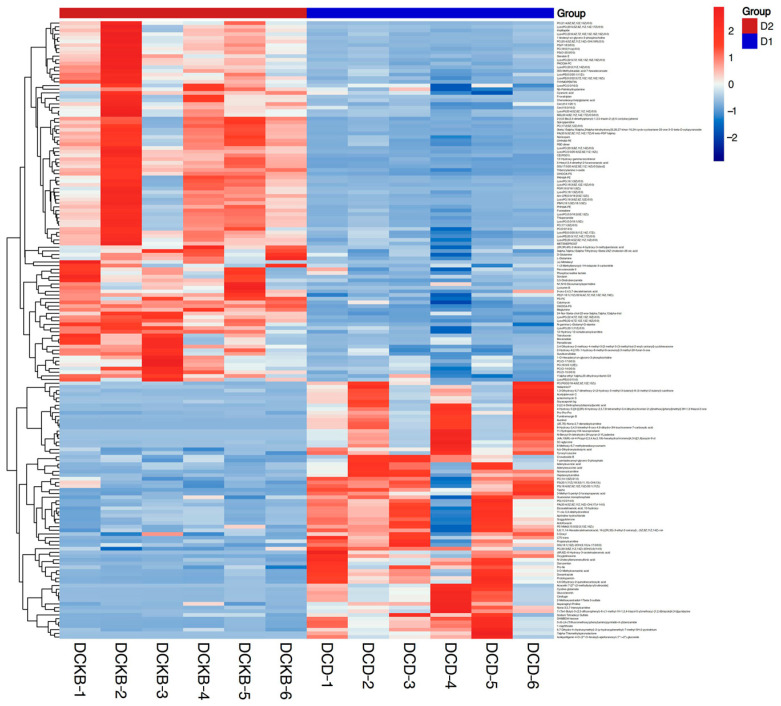
Heatmap of differential metabolites. Note: The horizontal coordinate represents the sample name, and the vertical coordinate represents the differential metabolite. The color from blue to red indicates that the expression abundance of the metabolite is from low to high; that is, the redder indicates the expression abundance of the differential metabolite.

**Figure 9:**
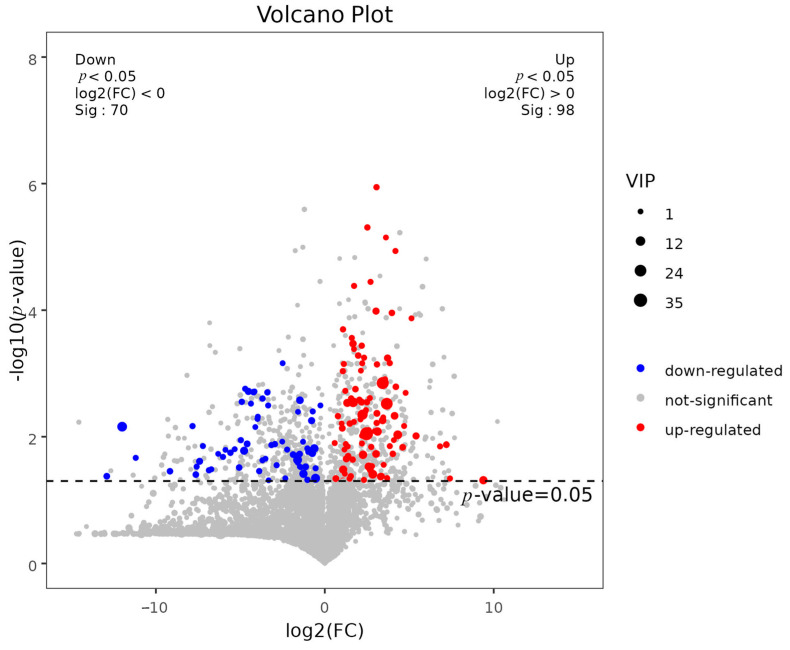
VIP values and screening of differential metabolite volcano map. Note: Each point in the figure represents a metabolite, with log_2_(FC) values for the two groups aligned in horizontal coordinates, −log_10_(*p*-value) values in vertical coordinates. Red dots represent significantly up-regulated metabolites (*p* < 0.05, VIP > 1, and FC > 1), blue dots represent significantly down-regulated metabolites (*p* < 0.05, VIP > 1 and FC < 1), and gray dots represent non-significantly different metabolites.

The method of combining multidimensional analysis and one-dimensional analysis was used to screen the different metabolites between groups. In OPLS-DA analysis, the variable important in projection (VIP) can be used to measure the influence of the expression pattern of each metabolite on the classification and discrimination of each group of samples. A *T*-test was used to verify the significance of the different metabolites between groups. As shown in Figure 8, a total of 168 differential metabolites were screened between the control and experimental groups. As can be seen in Figure 9, 98 differential metabolites were up-regulated and 70 differential metabolites were down-regulated in the experimental group compared with the control group.

#### 2.5.3. Significant Differential Metabolites

**Figure 10:**
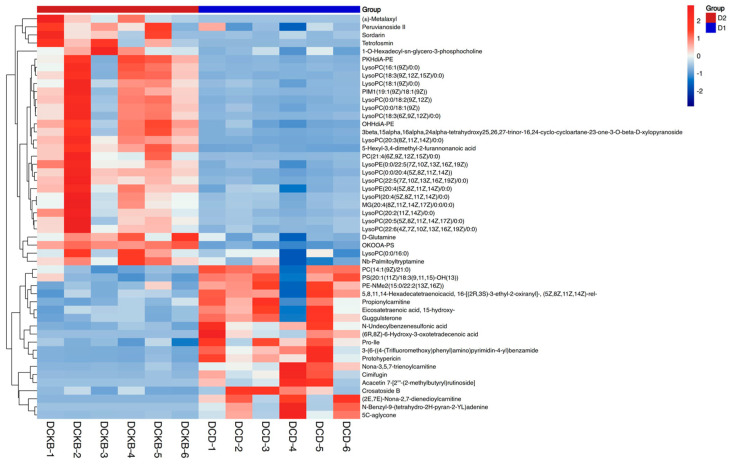
Heatmap of the top 50 differential metabolites.

To show the relationship between samples and the difference in the expression of metabolites between different samples, hierarchical clustering was performed on the expression levels of all significantly different metabolites and the significantly different metabolites ranked in the top 50 according to VIP, respectively, as shown in Figure 10. The top 15 metabolites with the most significant differences were selected for analysis, as shown in Table 6. This study also focused on the metabolites of *C. butyricum*. It was found that A, b-Dihydroxyisobutyric acid and 2-[(2,4-Dinitrophenyl) diazenyl] acetic acid were significantly down-regulated.

#### 2.5.4. Enrichment Analysis of Metabolic Pathways

**Figure 11:**
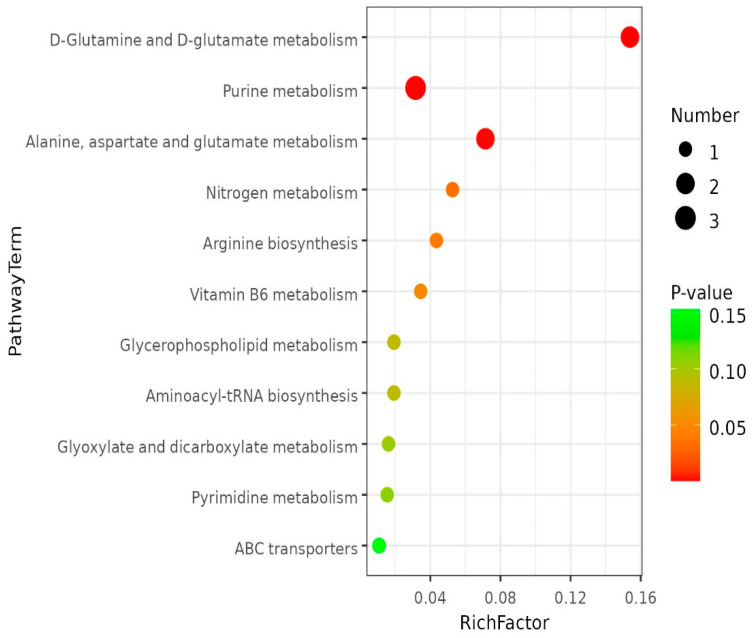
TOP-20 Enriched bubble map of metabolic pathway. Note: The vertical coordinate is the name of the metabolic pathway, and the horizontal coordinate is rich factor. The larger the rich factor is, the greater the enrichment degree. The color change from green to red indicates that the *p*-value decreases in sequence. The larger the point, the more metabolites are enriched in the pathway.

In this study, LC-MS metabonomics was used to analyze the intestinal samples of the two groups, and the difference between the two groups was statistically significant. Based on the KEGG metabolic pool, the metabolic pathways with significant differences in metabolites were identified. As shown in Figure 11, six metabolic pathways were preliminarily analyzed and selected, including D-glutamine and D-glutamate metabolism, purine metabolism, alanine, aspartate, and glutamate metabolism, nitrogen metabolism, arginine biosynthesis, and vitamin B6 metabolism.

### 2.6. Joint Analysis of Metabolomics and High-Throughput Sequencing

**Figure 12:**
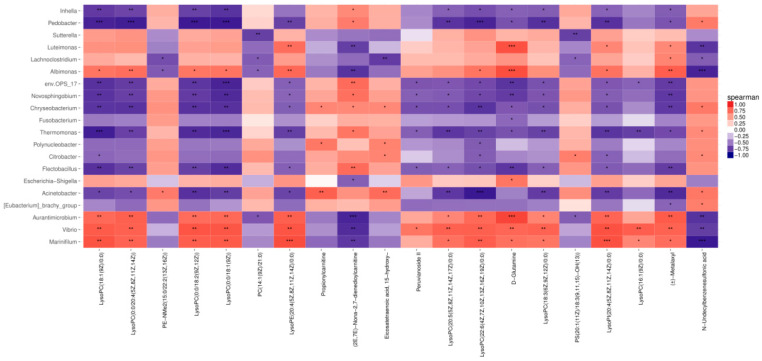
Correlation heat map of bacterial abundance and metabolites between control group and experimental group. Note: For each species that behaved differently and for each metabolite that was listed. The tangerine was positively correlated, the blue was negatively correlated, and the darker the color, the greater the correlation, and the closer the color to white, the closer the correlation to zero. *** in the figure represents correlation *p* < 0.001, ** in the figure represents correlation *p* < 0.01, and * in the figure represents correlation *p* < 0.05.

The effect of gut microbes on their host is realized by regulating metabolites. According to the sample-to-sample correspondence, the genera in the top 20 of the differential species and the metabolites in the top 20 of the differential metabolites were selected, and the Spearman correlation algorithm was used. The intrinsic association of the different metabolites between the control group and the test group with the microbial genus is shown in Figure 12. As can be seen from the graph, some differential metabolites in the significantly enriched pathway (*p* < 0.05) were significantly correlated with some genera.

## 3. Discussion

### 3.1. Effects of C. butyricum on Blood and Liver Antioxidant Capacity of E. sinensis 

Animals have an integrated antioxidant system in which enzymes play an important role in the antioxidant process, reflecting the ability of *E. sinensis* to resist stress [19]. Superoxide superoxide dismutase (SOD) and glutathione peroxidase (GSH-PX) are major antioxidant enzymes that convert superoxide anion radicals into oxygen and hydrogen peroxide, an adjustable balance between body oxidation and antioxidation [20], which removes excess damaging reactive oxygen species (ROS) and is an important component of the antioxidant defense system, and protect cells from damage caused by ROS through lipid peroxidation [21,22,23]. Catalase (CAT) is a good scavenger for the removal of H_2_O_2_, which has certain prevention and control effects on oxidative damage in the body [24]. Moreover, the activity of free radicals can reflect the degree of free radical removal in the body to some extent, so it is often regarded as a major indicator of the ability to eliminate free radicals [25,26]. Malondialdehyde (MDA) is used as a biomarker of tissue lipid peroxidation that can reflect the extent of oxidative damage in vivo, and high levels of MDA lead to high cytotoxicity [27]. The total antioxidant capacity (T-AOC) is divided into an antioxidant enzyme system and a non-enzymatic system in vivo, and is a comprehensive indicator of the body’s antioxidant capacity, with its main role being to decompose and scavenge reactive oxygen species (ROS) produced during the body’s metabolism [28,29].

The results showed that adding *C. butyricum* to the diet could increase the activities of CAT, GSH, T-AOC, and SOD in the *E. sinensis* blood and hepatopancreas and decrease the activity of MDA. This is consistent with the results obtained by using *C. butyricum* to feed young *Oreochromis aureus* [30]. This suggests that *C. butyricum* can enhance the antioxidant capacity of *E. sinensis* blood and hepatopancreas, enhancing their resistance to oxidative damage. It is well known that the hepatopancreas is an important organ for *E. sinensis* digestion, nutrient absorption and storage, fat and carbohydrate metabolism, and detoxification [31,32]. There is a strong correlation between antioxidant capacity and *E. sinensis* health. Therefore, adding a certain amount of *C. butyricum* can improve the health of *E. sinensis*.

### 3.2. Effects of C. butyricum on Intestinal Tissue of E. sinensis

The aquatic animal gut is not only an important organ for the digestion and absorption of nutrients but also the most important place for material and information exchange between the body and the outside world. It plays an important role in the immune system of the body; the integrity of intestinal morphology and function underlies normal digestion and absorption [33]. Intestinal villi play an important role in the digestion and absorption of nutrients, and the longer the villi are, the larger the area of the absorption site and the stronger the digestion capacity [34]. Crypt depth reflects the ability of intestinal cells to differentiate into villi [35,36]. In this study, the villus height and crypt depth in the experimental group showed an upward trend compared with those in the control group, indicating that *C. butyricum* can improve the *E. sinensis* intestinal tissue and improve the digestion and absorption abilities of the body.

### 3.3. Effects of C. butyricum on Intestinal Flora of E. sinensis

Probiotics, a group of living bacteria that play a beneficial role by improving the intestinal microflora of their host, can improve the structure of the intestinal balance of nature and regulate the disturbance of the intestinal flora caused by antibiotics or other factors, helping restore homeostasis [37]. At the same time, many studies have found that probiotics can improve the intestinal flora of *E. sinensis*, improve their antioxidant capacity, and enhance the immune system. For example, studies by Cao Haipeng et al. have shown that the nitrogen-fixing *Rhodobacter azotoformans* SY5 improves *E. sinensis* growth performance, immunity, antioxidant capacity, and intestinal health. The composition of the intestinal flora is an important component of the health of aquaculture animals. A rich and diverse gut microbiota composition promotes nutrient metabolism, immune response, and disease resistance in aquatic animals [38]. In this study, *C. butyricum* was added to the diet of *E. sinensis* and combined with high-throughput sequencing to study the effects of *C. butyricum* on the intestinal flora of *E. sinensis*.

The results showed that the microbial diversity and abundance of the intestinal flora in the *E. sinensis* of the experimental group with the addition of *C. butyricum* in the feed were significantly increased compared with the control group. Yuan Hailin et al. similarly found that the addition of a certain amount of *C. butyricum* to feed positively promoted the abundance and diversity of the gut microbiota of *Litopenaeus Vannamei*. In this experiment, the results of the sequencing of the intestinal flora in the experimental group and the control group showed that the dominant intestinal flora at the phyla level were *Firmicutes*, *Bacteroidetes*, and *Proteobacteria*, respectively. This suggests that these phyla play a significant role in digestion, absorption, and immune response in the gut of *E. sinensis*. Compared with the control group, the abundance of *Firmicutes* in the experimental group was significantly higher than that in the control group, while the abundance of *Proteobacteria* in the experimental group was lower than that in the control group, which was similar to the study of Yang Keng et al. [39]. This suggests that *C. butyricum* can regulate the abundance of *E. sinensis* intestinal flora and protect the intestinal health of farmed animals. Some species of the genus *Vibrio* are known to cause biological damage as well as disease, and a study found that the genus *Vibrio* was detected in the gut of *Litopenaeus Vannamei* with acute hepatopancreatic necrosis [40]. At the genus level, we found that the *Vibrio* and *Marinifilum* in the experimental group were significantly lower than those in the control group, which indicated that *C. butyricum* could decrease the *Vibrio* content, improve the body’s immune function, and promote the growth of *E. sinensis*. This is similar to the findings of Sumon et al., who showed that *C. butyricum* can reduce the amount of *Vibrio* in the *Macrobrachium rosenbergii* gut and attenuate its growth [41].

### 3.4. Effects of C. butyricum on Intestinal Flora Metabolism of E. sinensis

The metabolites detected in the *E. sinensis* intestinal flora were mainly lipids and lipid-like molecules. These lipid metabolites were mainly glycerophospholipids and fatty acids. This suggests that lipid-related metabolism is strongest in the *E. sinensis* gut. Glycerophospholipid is the most common phospholipid, which is involved not only in the construction of biofilms but also in the recognition and signaling of proteins by cell membranes [42]. In this study, lysophosphatidylcholine (LysoPC), which is concentrated in the glycerophospholipid, was the main differential metabolite and showed up-regulation. Lysophosphatidylcholine is the main active component of oxidationally modified low-density lipoprotein, which stimulates the expression of endothelial growth factors and adhesion molecules [43,44]. The lysophosphatidylcholine level was significantly up-regulated in the experimental group compared with the control group, indicating that the addition of *C. butyricum* to the diet could improve the immune function of *E. sinensis* and promote the absorption of nutrients. However, phosphatidylcholine (PC) was found to be down-regulated, presumably because *C. butyricum* promotes the breakdown of phosphatidylcholine and the expression of growth factors and adhesion molecules in endothelial cells. 

The pathways of KEGG enrichment were D-glutamine and D-glutamate metabolism, purine metabolism, alanine, aspartate, and glutamate metabolism, nitrogen metabolism, arginine biosynthesis, and vitamine B6 metabolism, respectively. Purine metabolism and alanine, aspartate, and glutamate metabolism pathways showed a downward trend. Reports suggest that an increase in purines within an organism predisposes the organism to a disturbed water-salt balance of cells, causing a foreign body in the tissue to interact with the joint inflammatory responses [45,46]. The results indicated that the addition of *C. butyricum* to feed could decrease purine metabolism, promote the balance of water and salt in organismal cells, and reduce inflammatory reactions. Studies by Wang Guiqin et al. have shown that vitamin B6 can promote feed utilization of *Channa argus*, enhance the activity of protein metabolic enzymes, promote protein metabolism, and then promote growth [47]. As a growth-essential amino acid, arginine can promote the synthesis of proteins in the body, which has important biological significance [48]. This study showed that the addition of *C. butyricum* increased the production of arginine, which in turn increased the body’s essential amino acid and growth.

In addition, a significant association was found between the *E. sinensis* gut microbiota and some of the different metabolites in the joint analysis. When the number of *Vibrio* in the intestine increased, the metabolite of lysophosphatidylcholine was up-regulated. Phosphatidylcholine is mainly concentrated in the glycerophospholipid pathway; glycerophospholipid metabolic pathways have been implicated in the immunomodulatory system and cellular inflammatory responses [49,50,51]. Therefore, it can be inferred that *C. butyricum*, in the presence of *Vibrio* species, can affect the up-regulation of lysophosphatidylcholine in the glycerophospholipid metabolic pathway, thereby enhancing the immune capacity of the *E. sinensis* organism and reducing the harm to the body. However, its specific mechanism is still a little vague and need to be further explored. In conclusion, *C. butyricum* can regulate the growth performance of *E. sinensis* by regulating the structure of the intestinal flora and then affecting the up-regulation or down-regulation of metabolites.

## 4. Materials and Methods

### 4.1. Strain and Feed Preparation

The pure bacterial solution of *C. butyricum* used in this experiment was provided by Huijia Biotechnology Co., Ltd., Anji 313306, China. The number of viable bacteria after resuscitation and expansion was 1 × 10^10^ CFU/g. *C. butyricum* was added to the basal diet, and the number of viable bacteria in the diet was 0 CFU/g, 1 × 10^6^ CFU/g, and 1 × 10^8^ CFU/g, respectively. Each treatment was referred to as the control group, experimental group 1, and experimental group 2.

### 4.2. Laboratory Animal

The *E. sinensis* was raised temporarily for 7 days. After the end of the temporary feeding period, 81 healthy crabs of uniform size were selected and randomly divided into 3 groups, with 3 replicates per group and 9 crabs per replicate. It is cultured in an aquarium (77 × 53 × 50 cm) with the same environmental conditions. Three experimental diets were fed four times a day. Continuous culture for 28 days, during which water, sewage, and oxygenation will be carried out from time to time. 

### 4.3. Determination of Serum Antioxidant Capacity

After the end of breeding, nine crabs were randomly selected from the control group, experimental group 1, and experimental group 2, respectively. After anesthesia on ice, a 1 mL sterile syringe was used to extract hemolymph from the base of the third step foot, and the hemolymph samples were stored in an ultra-low-temperature refrigerator at −80 °C. The thawed hemolymph was homogenized and centrifuged (12,000 r/min, 4 °C for 20 min). The content of the serum antioxidant index was determined according to the instructions of the kit.

### 4.4. Determination of Digestive Enzymes and Antioxidant Capacity of Hepatopancreas

The above 9 crabs were taken out of the hepatopancreas, quickly placed in liquid nitrogen, and then stored in a refrigerator at −80 °C for detection. The antioxidant indexes were catalase (CAT), glutathione peroxidase (GSH), superoxide dismutase (SOD), malondialdehyde (MDA), and total antioxidant capacity (T-AOC). All of them were determined by the kit of Biyuntian Biotechnology Co., Ltd., Shanghai 201611, China and the determination method was carried out according to the instructions.

### 4.5. Observation on Intestinal Morphology of E. sinensis

Six crabs were taken from the control group, experimental groups 1 and 2, respectively, dissected and removed the intestine, fixed in Bouin’s solution for 24 h, and stored in 75% ethanol for later use. After ethanol dehydration, paraffin embedding, sectioning, hematoxylin-eosin staining, and neutral gum sealing, the fixed tissues were observed and photographed under a microscope.

### 4.6. Determination of Intestinal Flora of E. sinensis

#### 4.6.1. Sample Collection

According to the results of serum and hepatopancreatic antioxidant indexes measured from *E. sinensis*, and based on the changes in intestinal morphology, intestinal flora, and metabolomics analysis, we only selected 12 samples from the control group and experimental group 2 for determination and analysis. After the end of culture, six crabs were selected from each group, and the intestine was dissected and divided into two parts: one for the determination of intestinal flora and one for LC-MS non-targeted metabolomics analysis.

#### 4.6.2. Total DNA Extraction and PCR Amplification of Intestinal Flora

The intestinal samples of *E. sinensis* were quickly frozen and stored at −80 °C. Intestinal DNA was extracted using the DNeasy PowerSoil kit. DNA concentration and purity were determined by the NanoDrop 2000 spectrophotometer and agarose gel electrophoresis, respectively. The extracted DNA was used as a PCR template to amplify the hypervariable V3-V4 region of the 16S ribosomal RNA gene. The upstream primer was 343F: 5′-TACGGRAGGCAGCAG-3′, and the downstream primer was 798R: 5′-AGGGTATCTAATCCT-3′. Two rounds of PCR amplification were performed. The reaction system and reaction parameters are shown in Tables S1–S4.

#### 4.6.3. Purification and Quantification of PCR Products

The quality of Amplicon was visualized by gel electrophoresis. The PCR products were purified by Agencourt AMPure XP beads and quantified by Qubit dsDNA assay kit, and then the concentration was adjusted for sequencing.

#### 4.6.4. Sequencing Data Processing and Analysis of Intestinal Microorganisms

The original sequencing data was in FASTQ format. Firstly, cutadapt (version: 2020.11.1) software is used to preprocess the raw data sequence. Then, using DADA2 (version: 2020.11.1), the qualified two-terminal raw data in the previous step is subjected to quality control analysis such as quality filtering, noise reduction, splicing, and chimera removal according to the default parameters of QIIME 2 (version: 2020.11.1) [52,53], and the representative sequence and ASV abundance table are obtained. After selecting the representative sequences of each ASV using the QIIME 2 software package, all representative sequences were compared and annotated with the Silva (version 138) database. The species alignment annotation is analyzed using the default parameters of the q2-feature-classifier software. Alpha diversity, including the Chao1 index and Shannon index, was used to estimate the microbial diversity in the intestinal samples of *E. sinensis*. The unweighted Unifrac principal coordinate analysis (PCoA) and phylogenetic tree construction were performed using the Unifrac distance matrix executed by QIIME software.

### 4.7. Study on the Metabolomics of Intestinal Flora

#### 4.7.1. Sample Pretreatment

Firstly, the treated sample of 30 mg was weighed and put into the EP tube; two small steel balls and 400 μL of methanol-water were added, pre-cooled for 2 min, and then ground. Then, in the ice water bath for 10min and then standing for 30 min, centrifuge (13,000 rpm, 4 °C, 10 min), take 300 μL of supernatant, and put it into an LC-MS injection vial. Then redissolve and let stand for 2 h. Finally, centrifuge for 10 min (13,000 rpm, 4 °C), extract 150 μL supernatant with a syringe, filter with a 0.22 μm organic phase pinhole filter, transfer to an LC sample vial, and store at −80 °C until LC-MS analysis.

#### 4.7.2. Chromatographic Mass Spectrometry Conditions

The analytical instrument of this experiment was a liquid chromatography-mass spectrometry system composed of an ACQUITY UPLC I-Class plus ultra-high performance liquid chromatography tandem QE high-resolution mass spectrometer. The chromatographic column was ACQUITY UPLC HSS T3 (100 mm × 2.1 mm, 1.8 μm), and the column temperature was maintained at 45 °C. The mobile phases were A-water (containing 0.1% formic acid) and B-acetonitrile, and the flow rate was maintained at 0.35 mL/min. The injection volume was 5 μL. An ESI ion source was used, and the sample mass spectrometry signal acquisition was performed in positive and negative ion scanning modes. The test conditions are shown in Tables S1–S4.

### 4.8. Analysis of Data

Statistical software SPSS 23.0 was used to perform a one-way analysis of variance (ANOVA) on the obtained data. The experimental data were expressed as mean ± standard error, and the significance level was set to (*p* < 0.05).

## 5. Conclusions

The results showed that the addition of *C. butyricum* to the diet increased the Hapten ability of *E. sinensis* serum and hepatopancreas and decreased the activity of malondialdehyde (MDA). At the same time, it increases the villi height of intestinal tissue and has a protective effect on it. In addition, *Vibrio*, *Bacteroides*, and *Shivella* were found to be inhibited at the *E. sinensis* level. The metabolism of intestinal microbes was analyzed by metabolomics, and it was found that lysophosphatidylcholine was significantly up-regulated in the experimental group with the addition of *C. butyricum*. Its metabolic pathways are mainly involved in D-glutamine and D-glutamate metabolism, purine metabolism, alanine, aspartate, and glutamate metabolism, nitrogen metabolism, arginine biosynthesis, and vitamin B6 metabolism. In conclusion, *C. butyricum* can inhibit the pathogenic bacteria in the *E. sinensis* gut and improve intestinal health and growth performance. These results provide a more theoretical basis for the action pathway and application effect of *Clostridium butyricum* in the *E. sinensis*. This also provides a theoretical basis for the further development of *Clostridium butyricum* and its application in aquatic animal culture. 

## Figures and Tables

**Table 1:** Effects of different concentrations of *C. butyricum* on serum antioxidant indices of *E. sinensis*.

Item	Control Group	10^6^ cfu/mL	10^8^ cfu/mL
CAT (U/mL)	4.0119 ± 0.3165 ^a^	4.2685 ± 0.3923 ^a^	4.4195 ± 0.5387 ^a^
GSH (U/mg)	0.6802 ± 0.1658 ^a^	0.7851 ± 0.1148 ^b^	0.8446 ± 0.076 ^b^
MDA (mU/mL)	0.0296 ± 0.0147 ^a^	0.0266 ± 0.0081 ^a^	0.0124 ± 0.0057 ^b^
SOD (U/mg)	61.0360 ± 1.9820 ^a^	58.5744 ± 2.2518 ^b^	61.3155 ± 1.8405 ^a^
T-AOC (U/mg)	0.2385 ± 0.0182 ^a^	0.2086 ± 0.0357 ^a^	0.2409 ± 0.0526 ^a^

Note: Values with different superscript letters in the same column are statistically significant (*p* < 0.05).

**Table 2:** Effects of different concentrations of *C. butyricum* on antioxidant indexes in hepatopancreas of *E. sinensis*.

Item	Control Group	10^6^ cfu/mL	10^8^ cfu/mL
CAT (U/mL)	0.3475 ± 0.0444 ^a^	0.3630 ± 0.0418 ^a^	0.4294 ± 0.0715 ^b^
GSH (U/mg)	0.5940 ± 0.0607 ^a^	0.5791 ± 0.0386 ^a^	0.6530 ± 0.0179 ^b^
MDA (mU/mL)	0.4645 ± 0.3984 ^a^	0.4217 ± 0.1647 ^a^	0.4146 ± 0.1290 ^a^
SOD (U/mg)	16.8892 ± 11.8846 ^a^	15.8798 ± 4.4397 ^a^	17.6362 ± 3.9137 ^a^
T-AOC (U/mg)	0.0744 ± 0.0285 ^a^	0.0817 ± 0.0380 ^a^	0.1134 ± 0.0389 ^a^

Note: Values with different superscript letters in the same column are statistically significant (*p* < 0.05).

**Table 3:** Effects of *C. butyricum* on intestinal microstructure indexes of *E. sinensis*.

Item	Villus Length (μm)	Crypt Depth (μm)
control group	154.19 ± 34.24 ^a^	61.91 ± 14.90 ^a^
10^6^ cfu/mL	189.24 ± 23.81 ^a^	76.52 ± 8.90 ^a^
10^8^ cfu/mL	214.13 ± 11.51 ^b^	104.24 ± 25.39 ^b^

Note: Values with different superscript letters in the same column are statistically significant (*p* < 0.05).

**Table 4:** Statistics of sequencing information of each sample.

Sample-ID	Raw_Reads	Filtered	Percentage of Input Passed Filter	Goods_Coverage	ASV_Counts
CD-1	81,936	75,018	91.56	0.99997	138
CD-2	80,091	77,197	96.39	0.999902	238
CD-3	80,954	77,647	95.91	0.999859	394
CD-4	80,971	76,959	95.05	0.99993	268
CD-5	80,137	74,716	93.24	0.999894	222
CD-6	78,815	75,950	96.36	0.999924	334
CKB-1	80,713	62,144	76.99	1	119
CKB-2	80,897	77,519	95.82	0.99992	184
CKB-3	81,527	76,531	93.87	0.9999	170
CKB-4	79,677	76,824	96.42	0.999893	274
CKB-5	78,223	74,946	95.81	0.999928	138
CKB-6	81,147	77,416	95.4	0.999906	242

**Table 5:** Richness and diversity indices of gut microbiota in *E. sinensis* treated with *C. butyricum*.

Concentration	Alpha Diversity Index		
(CFU/g)	Ace	Chao	Shannon
0	188.59 ± 59.54 ^a^	188.24 ± 59.87 ^a^	3.38 ± 0.46 ^a^
1 × 10^8^	266.43 ± 89.66 ^a^	266.09 ± 89.68 ^a^	4.43 ± 0.27 ^b^

Note: Values with different superscript letters in the same column are statistically significant (*p* < 0.05).

**Table 6:** The difference of the first 15 metabolites between control group and experimental group.

Metabolites	VIP	*p*-Value	log2(FC)	FC	Trend
LysoPC (18:1(9Z)/0:0)	32.861	0.00897	2.47068	5.54306	↑ **
LysoPC (0:0/20:4(5Z,8Z,11Z,14Z))	26.4308	0.00142	3.42823	10.7646	↑ **
LysoPC (0:0/18:2(9Z,12Z))	24.3984	0.00301	3.66833	12.7139	↑ **
LysoPC (0:0/18:1(9Z))	15.3216	0.00448	2.23524	4.7084	↑ **
(2E,7E)-Nona-2,7-dienedioylcarnitine	13.108	0.00694	−11.998	0.00024	↓ **
LysoPE (20:4(5Z,8Z,11Z,14Z)/0:0)	12.7626	0.00285	1.67581	3.19499	↑ **
PE-NMe2 (15:0/22:2(13Z,16Z))	10.0736	0.04522	−0.5477	0.68411	↓ *
Propionylcarnitine	9.2894	0.02319	−1.6108	0.32742	↓ *
LysoPC (18:3(6Z,9Z,12Z)/0:0)	9.06324	0.00932	4.31848	19.9522	↑ **
LysoPC (20:5(5Z,8Z,11Z,14Z,17Z)/0:0)	8.99216	0.03905	2.84103	7.16533	↑ *
LysoPC (22:6(4Z,7Z,10Z,13Z,16Z,19Z)/0:0)	7.70625	0.01928	2.26058	4.79186	↑ *
LysoPC (16:1(9Z)/0:0)	7.68579	0.00828	3.12461	8.72169	↑ **
PC (14:1(9Z)/21:0)	7.64072	0.01529	−0.622	0.64975	↓ *
LysoPI (20:4(5Z,8Z,11Z,14Z)/0:0)	7.22517	0.02954	2.60313	6.07602	↑ *
Eicosatetraenoic acid, 15-hydroxy-	6.98094	0.03844	−1.2653	0.41602	↓ *

Note: “**” in the table represents the trend of differential metabolites *p* < 0.01, and “*” in the table represents the trend of differential metabolites *p* < 0.05.

## Data Availability

The original contributions presented in the study are included in the article/Supplementary Material; further inquiries can be directed to the corresponding authors.

## Supplementary Materials

The following supporting information can be downloaded at: https://www.mdpi.com/article/10.3390/ijms241813784/s1.

## References

[1] Gu X., Zhao F. (2001). Resources and Culturing Situation of Chinese Mitten Crab (*Eriocheir sinensis*) and Species Character Conservation. J. Lake Sci..

[2] Ding Z. (2023). Current Disease Threats for Cultivated Crab *Eriocheir sinensis* in China. Transbound. Emerg. Dis..

[3] Luis Balcazar J., Rojas-Luna T., Cunningham D.P. (2007). Effect of the addition of four potential probiotic strains on the survival of pacific white shrimp (*Litopenaeus vannamei*) following immersion challenge with *Vibrio parahaemolyticus*. J. Invertebr. Pathol..

[4] Wang Y.-B., Li J.-R., Lin J. (2008). Probiotics in aquaculture: Challenges and outlook. Aquaculture.

[5] Wan J.-J., Pan J.-L., Shen M.-F., Xue H., Sun M.-L., Zhang M.-Q., Zhu X.-H., Ma X.-K. (2022). Changes in the growth performance, antioxidant enzymes and stress resistance caused by dietary administration of synbiotic (fructooligosaccharide and probiotics) in juvenile Chinese Mitten Crab, *Eriocheir sinensis*. Aquac. Int..

[6] Zhang C., Hou T., Yu Q., Wang J., Ni M., Zi Y., Xin H., Zhang Y., Sun Y. (2022). *Clostridium butyricum* improves the intestinal health of goats by regulating the intestinal microbial community. Front. Microbiol..

[7] Peng X., Tian Y., Ma X., Ge J., Huang Y. (2023). Effect of *Clostridium butyricum* on antioxidant, immunity function and digestive ability of *Eriocheir sinensis* juveniles. Feed Res..

[8] Cao H., Huang X., Gu Y., Zheng X., Xu L., Gai C. (2022). Protective effects of *Bacillus licheniformis* against *Citrobacter freundii* infection in Chinese mitten crab *Eriocheir sinensis*. J. Invertebr. Pathol..

[9] Fu J., Li L., Liu J., Liao T., Tie Y., Wen X. (2020). Research progress in the application of *Clostridium butyricum* and its metabolites in food processing. Food Ferment. Ind..

[10] Ma L., Shen Q., Lyu W., Lv L., Wang W., Yu M., Yang H., Tao S., Xiao Y. (2022). *Clostridium butyricum* and Its Derived Extracellular Vesicles Modulate Gut Homeostasis and Ameliorate Acute Experimental Colitis. Microbiol. Spectr..

[11] Nicholson J.K., Wilson I.D. (2003). Understanding ‘global’ systems biology: Metabonomics and the continuum of metabolism. Nat. Rev. Drug Discov..

[12] Liu C., Zhao L.-P., Shen Y.-Q. (2021). A systematic review of advances in intestinal microflora of fish. Fish Physiol. Biochem..

[13] Wu Y., Yang K., Huang X., Zhou C., Xu C., Huang Z., Yu W., Xun P., Huang J., Mai X. (2022). Effects of dietary *Clostridium butyricum* supplementation on growth performance and intestinal flora of juvenile *Trachinotus ovatus*. South China Fish. Sci..

[14] Duan Y., Wang Y., Dong H., Ding X., Liu Q., Li H., Zhang J., Xiong D. (2018). Changes in the Intestine Microbial, Digestive, and Immune-Related Genes of *Litopenaeus vannamei* in Response to Dietary Probiotic *Clostridium butyricum* Supplementation. Front. Microbiol..

[15] Yuan H., Li X., Sun Qn Tan X., Su Y., Huang Y., Yin W., Zhou M. (2023). Effects of *Clostridium butyricum* spores on growth performance, biochemical parameters in serum, intestinal flora and short-chain fatty acid content of *Paneaus vannamei*. J. South China Agric. Univ..

[16] Yamamoto M., Ohmori H., Takei D., Matsumoto T., Takemoto M., Ikeda M., Sumimoto R., Kobayashi T., Ohdan H. (2022). *Clostridium butyricum* affects nutrition and immunology by modulating gut microbiota. Biosci. Microbiota Food Health.

[17] Li P., Hou D., Zhao H., Wang H., Peng K., Cao J. (2022). Dietary *Clostridium butyricum* Improves Growth Performance and Resistance to Ammonia Stress in Yellow Catfish (*Pelteobagrus fulvidraco*). Aquac. Nutr..

[18] Zhu J., Wen B., Cheng G., Gao J., Chen Z. (2023). Effects of *Clostridium butyricum* on growth, body index, nutrient utilization, and intestinal structure and function of discus fish *Symphysodon haraldi*. J. Fish. China.

[19] Rengpipat S., Rukpratanporn S., Piyatiratitivorakul S., Menasaveta P. (2000). Immunity enhancement in black tiger shrimp (*Penaeus monodon*) by a probiont bacterium (*Bacillus S11*). Aquaculture.

[20] McCord J.M., Fridovich I. (1969). Superoxide dismutase. An enzymic function for erythrocuprein (hemocuprein). J. Biol. Chem..

[21] Yang G., Tian X., Dong S., Peng M., Wang D. (2015). Effects of dietary *Bacillus* cereus G19, B-cereus BC-01, and *Paracoccus marcusii* DB11 supplementation on the growth, immune response, and expression of immune-related genes in coelomocytes and intestine of the sea cucumber (*Apostichopus japonicus Selenka*). Fish Shellfish. Immunol..

[22] Shao X.-P., Liu W.-B., Xu W.-N., Lu K.-L., Xia W., Jiang Y.-Y. (2010). Effects of dietary copper sources and levels on performance, copper status, plasma antioxidant activities and relative copper bioavailability in *Carassius auratus gibelio*. Aquaculture.

[23] Zhou J., Ao X., Lei Y., Ji C., Ma Q. (2020). *Bacillus subtilis* ANSB01G culture alleviates oxidative stress and cell apoptosis induced by dietary zearalenone in first-parity gestation sows. Anim. Nutr..

[24] Chelikani P., Fita I., Loewen P.C. (2004). Diversity of structures and properties among catalases. Cell. Mol. Life Sci..

[25] Wang X.-J., Hu G.-C., Zhang L.i.-J., Li J.-Z., Guo S., Liu Y., Gan L., Cui K. (2016). Influence of cadmium on antioxidant defense system injuvenile of *Orechromis niloticus*. Mar. Environ. Sci..

[26] Sandamalika W.M.G., Kwon H., Lim C., Yang H., Lee J. (2021). The possible role of catalase in innate immunity and diminution of cellular oxidative stress: Insights into its molecular characteristics, antioxidant activity, DNA protection, and transcriptional regulation in response to immune stimuli in yellowtail clownfish (*Amphiprion clarkii*). Fish Shellfish Immunol..

[27] Pan J.-H., Feng L., Jiang W.-D., Wu P., Kuang S.-Y., Tang L., Zhang Y.-A., Zhou X.-Q., Liu Y. (2017). Vitamin E deficiency depressed fish growth, disease resistance, and the immunity and structural integrity of immune organs in grass carp (*Ctenopharyngodon idella*): Referring to NF-kappa B, TOR and Nrf2 signaling. Fish Shellfish Immunol..

[28] Xu M., Zhang J., Huang G., Zhang C., Cheng Y., Yang X. (2018). Effects of L-tryptophan and melatonin on the serum glucose level and antioxidant capacity in the hepatopancreas of Chinese mitten crab (*Eriocheir sinensis*). J. Fish. China.

[29] Lei Y., Sun Y., Wang X., Lin Z., Bu X., Wang N., Du Z., Qin J., Chen L. (2021). Effect of dietary phosphorus on growth performance, body composition, antioxidant activities and lipid metabolism of juvenile Chinese mitten crab (*Eriocheir sinensis*). Aquaculture.

[30] Qin M.-L., Hey-Z, Tan X.-Y., Zhang C.-Q., Liu Y.-Q., Meng L.-Q., Tong T., Zhang Q. (2022). Effect of *Clostridium butyricum* on growth performance, serum biochemical indexes and liver antioxidant indexes of juvenile tilapia. Feed Res..

[31] Zhang C., Wang X., Wang C., Song Y., Pan J., Shi Q., Qin J., Chen L. (2021). Gamma-aminobutyric acid regulates glucose homeostasis and enhances the hepatopancreas health of juvenile Chinese mitten crab (*Eriocheir sinensis*) under fasting stress. Gen. Comp. Endocrinol..

[32] Wang X., Wang L., Zhang H., Liu R., Song L. (2015). The carbohydrate metabolism of scallop *Chlamys farreri* in the immune response against acute challenge of *Vibrio anguillarum*. Aquac. Int..

[33] Purushothaman K., Tan J.K.H., Lau D., Saju J.M., Thevasagayam N.M., Wee C.L., Vij S. (2021). Feed Restriction Modulates Growth, Gut Morphology and Gene Expression in Zebrafish. Int. J. Mol. Sci..

[34] Huyben D., Chiasson M., Lumsden J.S., Pham P.H., Chowdhury M.A.K. (2021). Dietary Microencapsulated Blend of Organic Acids and Plant Essential Oils Affects Intestinal Morphology and Microbiome of Rainbow Trout (*Oncorhynchus mykiss*). Microorganisms.

[35] Liu J., Feng X., Zhang Y., Cao J. (2021). Differences in Intestinal Morphology and Enzymatic Characteristics between Kio and Goldfish in Hybrid and Self-Crossed Offsptings. Fish. Sci..

[36] Soares M.P., Cardoso I.L., Araujo F.E., De Angelis C.F., Mendes R., Mendes L.W., Fernandes M.N., Jonsson C.M., de Queiroz S.C.D.N., Duarte M.C.T. (2022). Influences of the alcoholic extract of *Artemisia annua* on gastrointestinal microbiota and performance of Nile tilapia. Aquaculture.

[37] Ma X., Wu X., Hu L. (2018). Effcets of Two Species of Dietary Viable Bacteria on Microbiota in Water and Intestinal of Juvenile Red Crucian Carp *Carassius auratus*. Fish. Sci..

[38] Li K., Guan W., Wei G., Liu B., Xu J., Zhao L., Zhang Y. (2007). Phylogenetic analysis of intestinal bacteria in the Chinese mitten crab (*Eriocheir sinensis*). J. Appl. Microbiol..

[39] Su H., McClarty G., Dong F., Hatch G.M., Pan Z.X.K., Zhong G.M. (2004). Activation of Raf/MEK/ERK/cPLA2 signaling pathway is essential for chlamydial acquisition of host glycerophospholipids. J. Biol. Chem..

[40] Anas A., Sukumaran V., Nampullipurackal Devarajan D., Maniyath S., Chekidhenkuzhiyil J., Mary A., Parakkaparambil Kuttan S., Tharakan B. (2021). Probiotics inspired from natural ecosystem to inhibit the growth of Vibrio spp. causing white gut syndrome in *Litopenaeus vannamei*. 3 Biotech.

[41] Madani N.S.H., Adorian T.J., Farsani H.G., Hoseinifar S.H. (2018). The effects of dietary probiotic Bacilli (*Bacillus subtilis* and *Bacillus licheniformis*) on growth performance, feed efficiency, body composition and immune parameters of whiteleg shrimp (*Litopenaeus vannamei*) postlarvae. Aquac. Res..

[42] Smith P., Owen D.M., Lorenz C.D., Makarova M. (2021). Asymmetric glycerophospholipids impart distinctive biophysical properties to lipid bilayers. Biophys. J..

[43] Perrin-Cocon L., Agaugue S., Coutant F., Saint-Mezard P., Guironnet-Paquet A., Nicolas J.F., Andre P., Lotteau V. (2006). Lysophosphatidylcholine is a natural adjuvant that initiates cellular immune responses. Vaccine.

[44] Nakano T., Raines E.W., Abraham J.A., Klagsbrun M., Ross R. (1994). Lysophosphatidylcholine upregulates the level of heparin-binding epidermal growth factor-like growth factor mRNA in human monocytes. Proc. Natl. Acad. Sci. USA.

[45] Liu H., Fu S., Qiu M., Lin M., Wang A., Ye J. (2019). The Effect of Lead (Pb) in Water on the Intestinal Structure and Function of Juvenile Grass Carp (*Ctenopharyngodon idellus*). J. South China Norm. Univ..

[46] Tingting L., Likun R., Dangfeng W., Minjie S., Qiuying L., Jianrong L. (2020). Effect of allicin and its mechanism of action in purine removal in turbot. J. Food Sci..

[47] Wang G., Niu X., Lu H., Li Z., Han Y., Zhao C. (2011). Effect of Dietary Energy and Vitamin B_6_ on Growth and Enzyme of Protein Metabolism in *Channa argus*. Acta Sci. Nat. Univ. Sunyatseni.

[48] Hoseini S.M., Khan M.A., Yousefi M., Costas B. (2020). Roles of arginine in fish nutrition and health: Insights for future researches. Rev. Aquac..

[49] Yang T., Hui R., Nouws J., Sauler M., Zeng T., Wu Q. (2022). Untargeted metabolomics analysis of esophageal squamous cell cancer progression. J. Transl. Med..

[50] Liu D., Wang J., Zeng H., Zhou F., Wen B., Zhang X., Luo Y., Wu W., Huang J., Liu Z. (2022). The metabolic regulation of Fuzhuan brick tea in high-fat diet-induced obese mice and the potential contribution of gut microbiota. Food Funct..

[51] Zhao L. (2012). UPLC-Q/TOF MS Analysis of GP Metabolism in RAW264.7 Cell Inflammation Model Interfering by NSAIDs. Master’s Thesis.

[52] Callahan B.J., McMurdie P.J., Rosen M.J., Han A.W., Johnson A.J.A., Holmes S.P. (2016). DADA2: High-resolution sample inference from Illumina amplicon data. Nat. Methods.

[53] Bolyen E., Rideout J.R., Dillon M.R., Bokulich N.A., Abnet C.C., Al-Ghalith G.A., Alexander H., Alm E.J., Arumugam M., Asnicar F. (2019). Reproducible, interactive, scalable and extensible microbiome data science using QIIME 2. Nat. Biotechnol..

