# Supplementary material for: Effects of Clostridium butyricum on Intestinal Microflora and Metabolism of Eriocheir sinensis
Source: Int J Mol Sci. 2023 Sep 7;24(18):13784. doi: 10.3390/ijms241813784 (PMC10531170; doi:10.3390/ijms241813784)
Supplement: Supplementary file 1 [file ijms-24-13784-s001.zip › ijms-2564172-supplementary.pdf]

Effects of *Clostridium butyricum* on intestinal microflora and metabolism of *Eriocheir sinensis*

Xiaoning Gao [a,b,1], *Xueting Liu* [a,b,1], *Yali Wang* [a,b], *Tianwei Wang* [a,b], *Di Fang* [a,b], *and Kun Hu* [a,b] *

[a] *National Demonstration Center for Experimental Fisheries Science Education, Shanghai Ocean University, Shanghai 201306, China*

[b] *National Pathogen Collection Center for Aquatic Animals, Shanghai Ocean University, Shanghai 201306, China*

***Correspondence**

Kun Hu, Shanghai Ocean University, Shanghai 201306, P. R. China.

E-mail addresses: khu@shou.edu.cn

[1] The first two authors contributed equally to this work.

Table S1 First round PCR reaction system

Name	Volume
2×Gflex PCR Buffer	15 μl
5 pmol/μl primer F	1 μl
5 pmol/μl primer R	1 μl
Template DNA	≥ 1 μl (50 ng)
Tks Gflex DNA Polymerase (1.25U/μl)	0.6 μl
H_2O	Make up to 30 μl
Total	30 μl

Table S2 First round PCR reaction parameters

Temperature	Time	Cycle Number
94℃	5min	1
94℃	30s	
56℃	30s	26
72℃	20s	
72℃	5min	1
4℃	hold	-

Table S3 Second round PCR reaction system

Name	Volume
2×Gflex PCR Buffer	15 μl
Tks Gflex DNA Polymerase (1.25U/μl)	0.6 μl
Adapter I5	1 μl
Adapter I7	1 μl
First product	Take 50ng
H_2O	Make up to 30 μl
Total	30 μl

Table S4 Reaction parameters of the second round of PCR

Temperature	Time	Cycle Number
94℃	5min	1
94℃	30s	
56℃	30s	7
72℃	20s	
72℃	5min	1
4℃	hold	-
